# Feasibility of Monitoring Heart and Respiratory Rates Using Nonwearable Devices and Consistency of the Measured Parameters: Pilot Feasibility Study

**DOI:** 10.2196/56547

**Published:** 2024-10-08

**Authors:** Kasumi Ikuta, Miya Aishima, Maiko Noguchi-Watanabe, Sakiko Fukui

**Affiliations:** 1 Department of Home Health and Palliative Care Nursing, Graduate School of Health Care Sciences, Tokyo Medical and Dental University, Bunkyo-Ku, Japan

**Keywords:** heart rate, older adults, respiratory rate, nonwearable devices, vital signs

## Abstract

**Background:**

As Japan is the world’s fastest-aging society with a declining population, it is challenging to secure human resources for care providers. Therefore, the Japanese government is promoting digital transformation and the use of nursing care equipment, including nonwearable devices that monitor heart and respiratory rates. However, the feasibility of monitoring heart and respiratory rates with nonwearable devices and the consistency of the rates measured have not been reported.

**Objective:**

In this study, we focused on a sheet-type nonwearable device (Safety Sheep Sensor) introduced in many nursing homes. We evaluated the feasibility of monitoring heart rate (HR) and respiratory rate (RR) continuously using nonwearable devices and the consistency of the HR and RR measured.

**Methods:**

A sheet-type nonwearable device that measured HR and RR every minute through body vibrations was placed under the mattress of each participant. The participants in study 1 were healthy individuals aged 20‐60 years (n=21), while those in study 2 were older adults living in multidwelling houses and required nursing care (n=20). The HR was measured using standard methods by the nurse and using the wearable device (Silmee Bar-type Lite sensor), and RR was measured by the nurse. The primary outcome was the mean difference in HR and RR between nonwearable devices and standard methods.

**Results:**

The mean difference in HR was −0.32 (SD 3.12) in study 1 and 0.04 (SD: 3.98) in study 2; both the differences were within the predefined accepted discrepancies (<5 beats/min). The mean difference in RR was −0.98 (SD 3.01) in study 1 and −0.49 (SD 2.40) in study 2; both the differences were within the predefined accepted discrepancies (3 breaths/min).

**Conclusions:**

HR and RR measurements obtained using the nonwearable devices and the standard method were similar. Continuous monitoring of vital signs using nonwearable devices can aid in the early detection of abnormal conditions in older people.

## Introduction

The vital signs of older adults change with age [1,2], making it necessary to monitor them at their residences and in nursing homes [3,4]. Continuous monitoring of their heart rate (HR) and respiratory rate (RR) to obtain essential data and immediate assessment of any abnormality by medical personnel have proven successful in reducing hospitalization and mortality rates [5]. However, measuring vital signs once or twice daily for older people living at home and in nursing homes is unrealistic [6]. Moreover, the impact of COVID-19 necessitated remote monitoring of vital signs with fewer visits from medical staff to prevent infections [7].

Therefore, continuous vital sign monitoring devices that can be used without affecting daily activities must be developed.

Wearable devices (WDs), including wrist-worn devices [8], ViSi Mobile (Sotera Wireless, Carlsbad, CA) [9], and health patches (VitalConnect, San Jose, CA) [9], can monitor vital signs without interfering with daily activities. Measuring vital signs continuously with a WD may improve patient comfort and safety [10-12] and reduce nurse workload [10] by reducing the number of nurse-conducted measurements. However, redness and itching are common among older adults using WDs [9] because their skin is particularly fragile and WDs come in direct contact with the skin [13]. Therefore, developing and introducing nonwearable devices (NWDs) for monitoring older patients continuously is desirable [14].

NWDs, which measure vital signs without direct patient contact, reduce older adults’ feeling of restraint and awareness of being monitored as well as the workload associated with vital sign assessments for care providers. NWDs such as highly sensitive fiber optic mattress [11,15] and office chairs [16] are being researched and developed. Verification of its use in magnetic resonance imaging as well as in medical and nursing care settings is underway [17] and has attracted widespread attention.

As Japan is the world’s fastest-aging society with a declining population [18], it is challenging to secure human resources for care providers [19]. Therefore, the Japanese government is promoting digital transformation and the use of nursing care equipment, including NWDs. Many nursing homes have purchased and introduced NWDs since 2015 when the government started providing assistance payment for such devices as policy guidance [20,21]. Sheet-type NWDs, such as Safety Sheep sensors α (NJI Co., Ltd., Fukushima, Japan), NEMURI SCAN (Paramount Bed Co., Ltd., Tokyo, Japan), and Mimamoleaf (Techno Horizon Co., Ltd., Nagoya, Japan), have been developed and introduced in Japan. We focused on Safety Sheep sensors α, which is a monitoring device that has been introduced in many nursing homes [22], including 3319 nursing homes in Japan as of December 2022. This NWD includes a highly sensitive pressure sensor placed under a mattress and does not come in contact with the individual’s skin. It detects body vibrations and calculates the individual’s status (lying on bed/moving on bed/getting out of bed), HR, and RR every minute. These data are displayed on a monitor at nursing homes. Care providers check these data and, if they are abnormal, can take immediate action and provide appropriate care.

However, the data from NWDs, including sheet-type NWDs, cannot be used by nursing homes as longitudinal data to evaluate changes in individuals accurately and provide appropriate care [23] because the consistency of the HR and RR measured by such instruments has not been evaluated yet [24]. Moreover, based on the accumulated data, it may be possible to detect deteriorating conditions during end-of-life care and unplanned hospital visits at an early stage, concentrate care on high-risk targets, and improve the quality of care using fewer personnel. Therefore, this study aimed to evaluate the feasibility of continuous HR and RR monitoring and consistency of the rates measured using the NWD.

## Methods

### Setting and Participants

This was a prospective, observational, and pilot feasibility study. Two studies were conducted to evaluate the feasibility of continuous HR and RR monitoring, and the consistency of the HRs and RRs was measured using an NWD (Safety Sheep sensors α). In this study, the study power was calculated based on a previous study [9], and a sample size of 20 was estimated to obtain sufficient data for analysis.

#### Study 1: Healthy Participants

Study 1 included 22 healthy participants aged 20‐60 years who were working at a company. The authors recruited participants from June 1 to June 30, 2022. Participants were excluded if they had been diagnosed with heart or respiratory disease. To maintain the privacy of the participants, their vital signs were measured in a private room in company A. The NWD was placed under the mattress after preparing a bed. Each participant came to the room at separate designated times for measurements.

#### Study 2: Older Adults Who Needed Nursing Care

Study 2 included 26 older adults aged ≥65 years who were living in multidwelling houses managed by a company and required nursing care. Each multidwelling house had a care worker who was available for 24 hours daily. Thus, older adults who required nursing care could immediately receive nursing care from their care workers. Managers working in multidwelling houses recruited older adults for this study from July 1 to October 30, 2022. Participants diagnosed with cardiac or respiratory disease or those with implanted medical electronic devices, such as pacemakers, were excluded. Each participant was visited at appointed dates and times, and their HR and RR were measured in their rooms.

### Data Collection

In this study, data were collected by 2 nurses: one measured the HR and RR, while the other recorded them. They waited for the participant to visit the private room at company A in study 1 but visited the participant’s room themselves in study 2. First, the nurses explained the purpose and methods of this study to the participants. Second, the participants lay on a bed with the NWD in place. Third, the nurse who took the measurements attached a WD to the participants’ chests and asked them to rest for 5 minutes. The same nurse then measured the RR over 1 minute and reported five sets of measurements to the recording nurse. The recording nurse documented the start time of the measurement and the number of RRs. After a 1-minute break, the HR was measured using the same method. After completing the measurements, the participants were informed that the procedure was complete, and the WD was removed. Finally, the participants were checked for skin abnormalities.

### Measurement

#### Nonwearable Device

**Figure 1.**
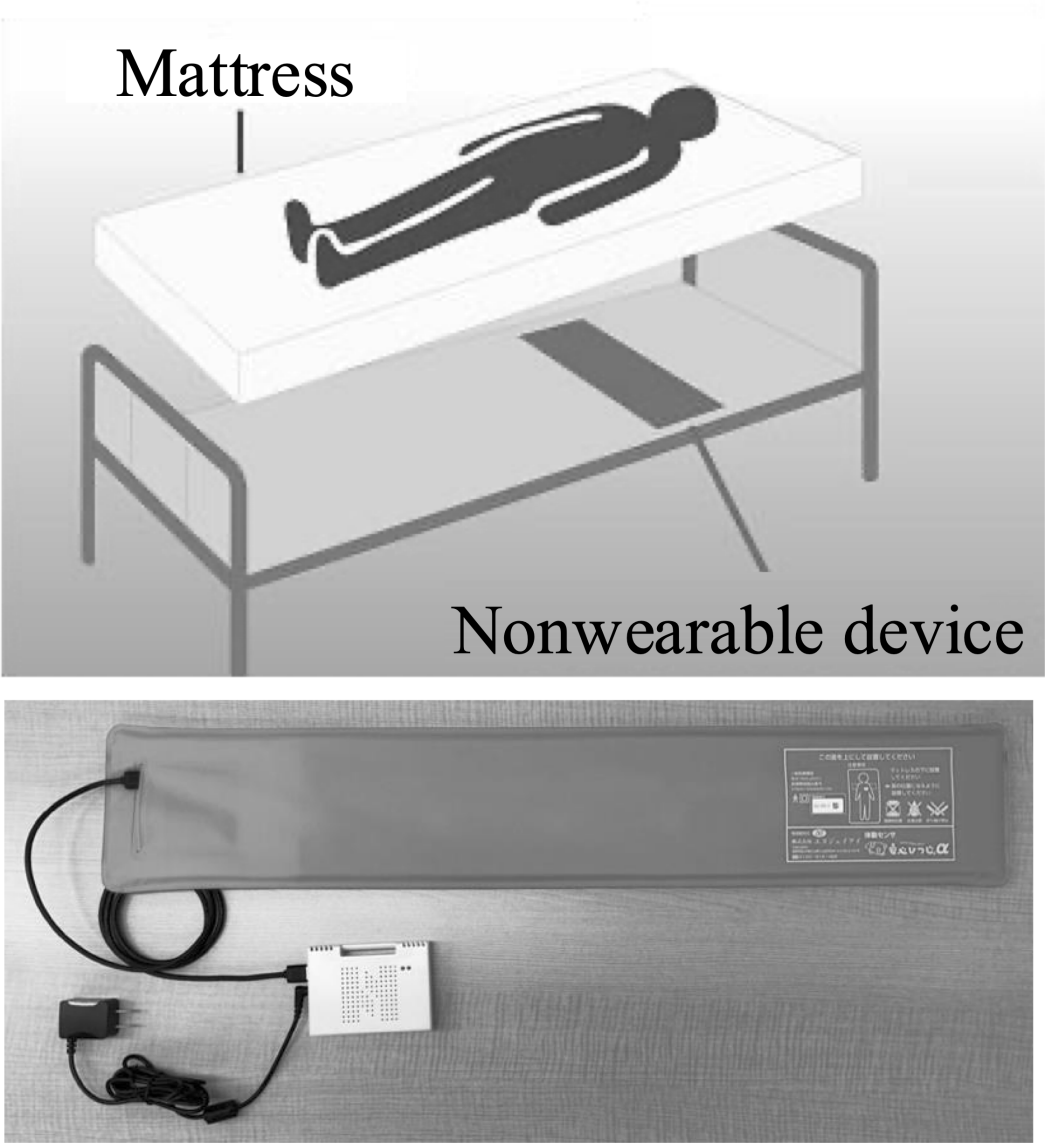
Positioning of the nonwearable devices.

**Figure 2.**
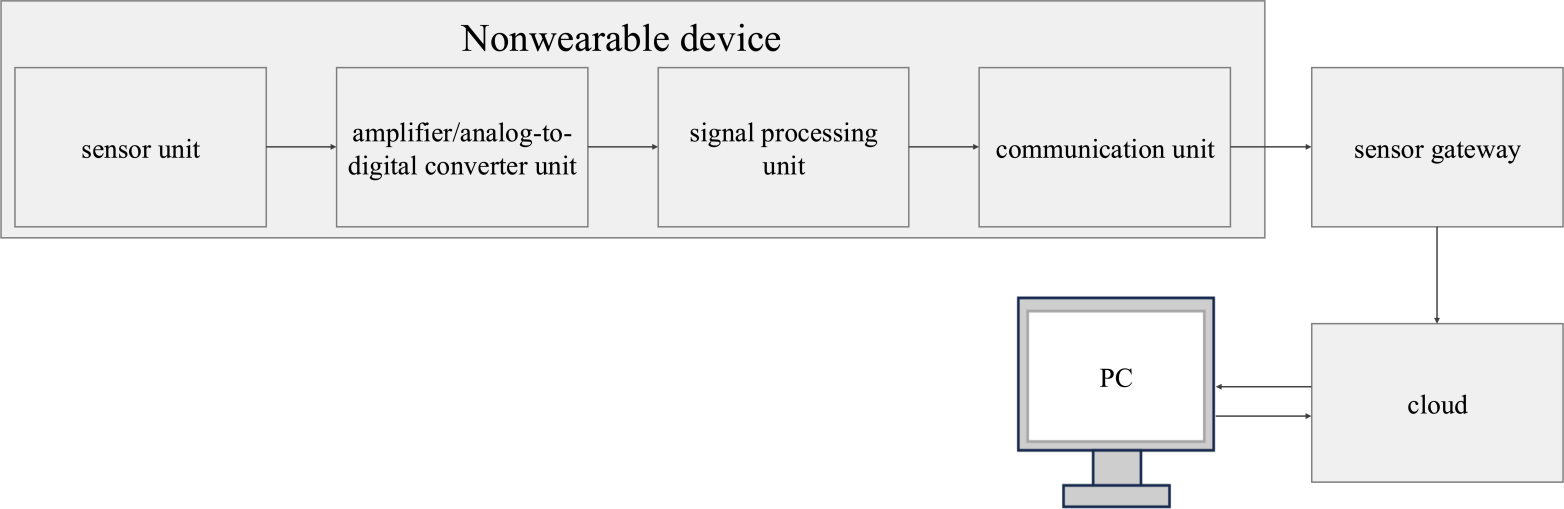
Nonwearable device system.

The Safety Sheep sensor α (width 800 mm; height 17 mm; depth 150 mm) was used as the NWD (Figure 1) [25]. In the multidwelling house, this NWD was placed in all the residents’ beds. The NWD comprises a sensor unit, amplifier/analog-to-digital converter unit, signal processing unit, and communication unit (Figure 2). The HR and RR are determined from heartbeat- and respiration-derived vibration waves transmitted through the device, respectively.

**Figure 3.**
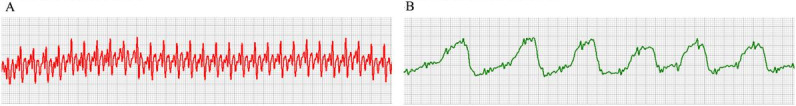
Heartbeat wave (A) and respiratory wave (B).

First, in the sensor unit, 6 piezoelectric elements capable of detecting minute vibrations are placed at 10-cm intervals. The vibrations generated by the participants mainly included those derived from heartbeat, respiration, and body movements. The vibration data acquired at the sensor section are converted into digital data at the amplifier/analog-to-digital converter unit. Subsequently, in the signal processing unit, HR and RR are determined from the digitally converted data (Figure 3).

Heartbeats have relatively high-frequency components and occur at periodic intervals; after passing through a high-pass filter, characteristics derived from heartbeats are extracted, and HR is detected from the intervals between the extracted characteristics. The frequency components of respiration mainly have low-frequency components and occur at relatively regular intervals; after passing through the low-pass filter, the same process used to detect HR is used to detect RR. The frequency components of body movement are mainly low-frequency components, like respiration, but often do not repeat cyclically, and the amplitude is much larger than that of respiration. Very large signals are generated simultaneously on several piezoelectric elements, distinguishing them from respiration. The determined HR and RR are sent via the communication unit and Raspberry Pi to the cloud. Care workers at the nursing homes can access the cloud server using a web application on their personal computers and view the data with a browser (Figure 2).

#### Measurement of Vital Signs by Nurses

In this study, the standard method used to measure HR and RR was performed by nurses as previously described [9,10,26]. Briefly, 2 nurses with 20 years of experience measured the HR and RR of all participants. The nurse measured the HR by touching the participant’s radial artery for 1 minute, and the RR by visually observing the thoracic movement for 1 minute. The nurse avoided touching the mattress while taking the measurements to eliminate interference.

Electrocardiograms and respiratory effort belts are other standard methods for measuring HR and RR; however, they are difficult to use in nursing homes staffed with only a few medical professionals [27,28]. Moreover, these contact devices have many electrodes and are not recommended for use in nonhospital settings as they cause physical restraint and discomfort in older adults [28]. Therefore, in this study, the standard method was used to ensure the safety of older adults and avoid causing discomfort.

#### Wearable Device

A Silmee Bar-type Lite sensor (TDK Co., Tokyo, Japan; width 64 mm; height 96 mm; depth 28 mm) was used as the WD to measure the HR of participants. This device could simultaneously measure electrocardiogram signals, pulse wave, acceleration, and skin temperature and was set to 125 Hz for the electrocardiograph and offline mode. This device was attached to the participants’ chests at 3 cm below the collarbone using a special gel pad, and their electrocardiogram signals were measured. The HR was calculated per minute based on the time between heartbeats, which is the device output from an electrocardiogram.

HR cannot be measured visually but is preferably measured at the location of the heart. Therefore, we measured the HR both by using the WD and by manually palpating the participant’s radial artery as mentioned in the previous section.

#### Collection of Basic Information

In study 1, the following items representing basic participant information were self-reported: sex, age, height, weight, respiratory diseases, and heart diseases. In study 2, the following data were collected from care workers in multidwelling houses: sex, age, height, weight, level of care needed, and diagnosis of dementia. Older adults who required nursing care were classified based on their care needs as levels 1‐5. Each municipality has certified care needs according to the level of nursing care required [29,30]. Care need level 1 requires relatively low assistance, while care need level 5 requires extensive assistance with personal care.

### Data Analysis

The primary outcome was the mean difference between the HR and RR measured using the NWD and those measured by the nurses and the WD. We defined HR<5 beats/min and RR<3 breaths/min as the accepted mean difference before starting the study. We also defined the maximum error not leading to a change in care or observation as the criterion based on previous studies [9,31].

HR and RR data from the NWD were each compared with measurements taken by the nurses and WD at the same time point. The measured data were compared at each time point, and measurement errors were calculated. Subsequently, Bland-Altman plots were created to assess the agreement between the data measured using the NWD and those measured using the WD [32,33]. This method has been widely used in comparative studies [32,34]. We calculated the mean difference and 95% limits of agreement (LoAs) between the NWD data and manually measured data. Python (version 3.8.1; Python Software Foundation) and R statistical software (version 3.6.3; R Foundation for Statistical Research) were used for analysis. All statistical tests were 2-sided, and statistical significance was set at *P*<.05.

### Ethical Considerations

This study was conducted in accordance with the principles of the Declaration of Helsinki and approved by the Human Study Ethics Committee of the authors’ affiliated university (approval number: M2021-374).

The participants were informed about the study aims and procedure, both verbally and in writing, and consented to participate by signing a consent form. For older adults with difficulty communicating due to dementia or other cognitive impairments, their relatives were contacted, and their proxy consent was obtained. All participants (and their family members, when needed) were informed that participation in the study was voluntary and that they could withdraw at any point. Moreover, we explained that the participants would not be disadvantaged by not participating in the study, discontinuing participation, or withdrawing their cooperation. To protect privacy and confidentiality, the data collection nurse assigned each participant an identification number, and the list linking names and identification numbers was kept by the nurse. The researchers did not have access to this list, ensuring that the data remained anonymized.

HR measurement using the WD required direct contact with the skin; therefore, we attempted to protect the skin by applying a protectant (PureBarrer; Granmate Co., Chiba, Japan) before attaching the device. After the measurements, a release agent (3M Cavilon Remover; 3M, Maplewood, MN) was used for careful removal. No compensation was provided to participants for their involvement in this study.

## Results

### Demographics

**Figure 4.**
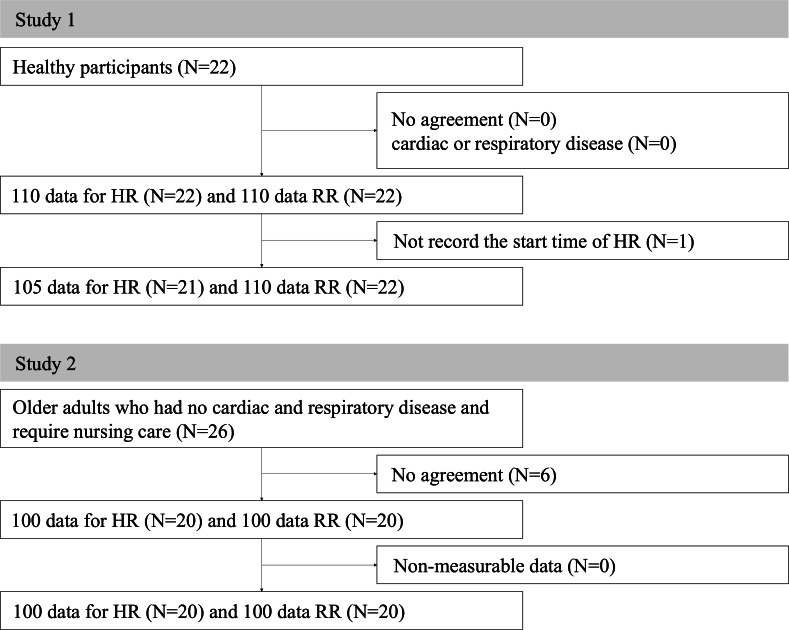
Flow diagram depicting the inclusion and exclusion of study participants.

A total of 22 healthy individuals participated in study 1 (Figure 4). The study population included 11 (50%) male participants and 11 (50%) female participants with a mean age of 47 (IQR 32.5‐57) years (Table 1).

**Table 1.** Participants’ characteristics for studies 1 and 2. Data are presented as median (IQR) or n (%).

	Value
**Study 1 (n=21)**	
Male sex, n (%)	11 (50)
Age (years), median (IQR)	47 (32.5‐57.0)
Body mass index (BMI; kg/m^2^), median (IQR)	22.2 (20.6‐23.9)
**Study 2 (n=20)**	
Male sex, n (%)	3 (15)
Age (years), median (IQR)	87.5 (85.0‐90.5)
BMI (kg/m^2^), median (IQR)	22.1 (20.1‐23.6)
Respiratory disease, n (%)	0 (0)
Care need level 1, n (%)	7 (35)
Care need level 2, n (%)	6 (30)
Care need level 3, n (%)	6 (30)
Care need level 4, n (%)	0 (0)
Care need level 5, n (%)	1 (5)
Dementia, n (%)	12 (60)

In study 2, 26 older adults requiring nursing care were selected, of whom 20 participated (Figure 4). Three older adults did not consent, and 3 could not obtain consent from their families. The study population included 3 (15%) male participants and 17 (85%) female participants with a mean age of 87.5 (IQR 85‐90.5) years (Table 1). Among the participants, 7 (35%) needed care level 1, 6 (30%) needed care level 2, 6 (30%) needed care level 3, and 1 (5%) needed care level 5. None of the participants dropped out during the study periods in studies 1 or 2.

### Technical Feasibility

In study 1, 110 HR data points (22 participants) and 110 RR data points (22 participants) were measured. The start time of HR measurement was not recorded for 1 participant owing to human error. Therefore, the NWD, WD, and nurse measurements for this participant could not be merged. We excluded the HR data of the participant (5 data points) and included 105 data points for HR (21 participants) and 110 data points for RR (22 participants).

In study 2, 100 data points each for HR (20 participants) and RR (20 participants) were measured. There were no missing data; therefore, 100 data points each were included for the HR (20 participants) and RR (20 participants).

### Heart Rate

The mean differences measured by the NWD, nurses, and WD are shown in Table 2. The mean HR in study 1 measured by the NWD was 66.57 (SD 8.45) beats/min, the nurse measured 66.25 (SD 8.05) beats/min, and the WD recorded 66.57 (SD 8.45) beats/min. The mean HRs in study 2 measured by the NWD, nurse, and WD were 64.22 (SD 6.13), 64.18 (SD 7.20), and 65.21 (SD 7.84) beats/min, respectively.

**Figure 5.**
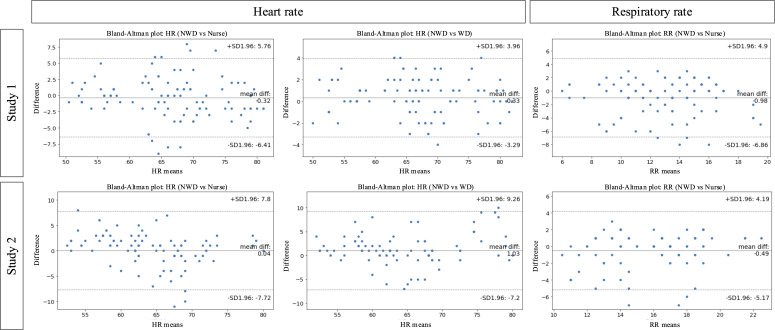
Bland-Altman plot. HR: heart rate; NWD: nonwearable device; RR: respiratory rate; WD: wearable device.

The Bland-Altman plots are shown in Figure 5 and Table 3. The mean, differences, and LoAs (1.96 SD) were plotted. The y-axis in Figure 5 indicates the measurement differences and LoAs. First, the differences between NWD and nurses for 95% were within the LoAs; however, wide LoAs were observed (study 1 [lower LoA to upper LoA]: −6.86 to 4.90; study 2: −7.72 to 7.80). Second, the differences between NWD and WD for 95% were within the LoAs; however, wide LoAs were observed (study 1: −3.42 to 4.02; study 2: −7.20 to 9.26).

The differences in the HR measurements are presented in Table 4. Approximately 90% of the measurement differences were within 5 measurements in studies 1 and 2.

**Table 2.** Means and differences of measurements taken using the NWD, nurse, and WD.

		Measurements, n	NWD^a^, mean (SD)	Nurse, mean (SD)	WD^b^, mean (SD)	NWD vs nurse, mean difference (SD)	NWD vs WD, mean difference (SD)
**Study 1**							
	HR^c^ (beats/min)	105	68.05 (6.9)	66.25 (8.05)	66.9 (8.28)	−0.32 (3.12)	0.33 (1.86)
	RR^d^ (beats/min)	110	13.27 (7.9)	12.55 (3.27)	—^e^	−0.98 (3.01)	—
**Study 2**							
	HR (beats/min)	100	66.13 (5.21)	64.22 (6.13)	65.21 (7.84)	0.04 (3.98)	1.03 (4.22)
	RR (beats/min)	100	23.22 (1.55)	15.65 (3.22)	—	−0.49 (2.4)	—

a NWD: nonwearable device.

b WD: wearable device.

c HR: heart rate.

d RR: respiratory rate.

e Not applicable.

**Table 3.** Bland-Altman analysis for HR and RR measured using the NWD.

		Measurements, n	NWD^a^ vs nurses			NWD vs WD^b^		
			Mean (SD)	Lower LoA^c^	Upper LoA	Mean (SD)	Lower LoA	Upper LoA
**Study 1**								
	HR^d^ (beats/min)	105	−0.32 (3.12)	−6.86	4.90	0.33 (1.86)	−3.42	4.02
	RR^e^ (beats/min)	110	−0.98 (3.01)	−6.41	5.76	—^f^	—	—
**Study 2**								
	HR (beats/min)	100	0.04 (3.98)	−7.72	7.80	1.03 (4.22)	−7.20	9.26
	RR (beats/min)	100	−0.49 (2.40)	−5.17	4.19	—	—	—

a NWD: nonwearable device.

b WD: wearable device.

c LoA: limit of agreement.

d HR: heart rate.

e RR: respiratory rate.

f Not applicable.

**Table 4.** Measurement difference for NWDs in studies 1 and 2.

		Study 1 measurements (HR^a^: n=105; RR^b^: n=110), n (%)		Study 2 measurements (HR and RR: n=100), n (%)	
		NWD^c^ vs nurse	NWD vs WD^d^	NWD vs nurse	NWD vs WD
**HR (beats/min)**					
	≤2	71 (67.62)	88 (83.81)	62 (62)	62 (62)
	>2	23 (21.9)	16 (15.24)	25 (25)	22 (22)
	>5	11 (10.48)	1 (0.95)	9 (9)	12 (12)
	>10	0 (0)	0 (0)	4 (4)	4 (4)
	>15	0 (0)	0 (0)	0 (0)	0 (0)
**RR (breaths/min)**					
	≤2	81 (73.64)	—^e^	84 (84)	—
	>2	19 (17.27)	—	11 (11)	—
	>5	9 (8.18)	—	5 (5)	—
	>10	0 (0)	—	0 (0)	—
	>15	1 (0.91)	—	0 (0)	—

a HR: heart rate.

b RR: respiratory rate.

c NWD: nonwearable device.

d WD: wearable device.

e Not applicable.

### Respiratory Rate

The mean differences measured by NWD, nurses, and WD are shown in Table 2. The mean RR in study 1 measured by the NWD was 13.54 (SD 3.31) breaths/min, and by the nurse was 12.55 (SD 3.27) breaths/min. The mean RR in study 2 measured by NWD was 16.14 (SD 2.89) breaths/min and by the nurse was 15.65 (SD 3.22) breaths/min.

The Bland-Altman plots are shown in Figure 5 and Table 3. The mean differences and LoAs (SD 1.96) were plotted. The y-axis in Figure 5 indicates the measurement differences and LoAs. The differences between the data obtained from the NWD and nurses for 95% were within the LoAs. However, wide LoAs were observed (study 1 [lower LoA to upper LoA]: −6.41 to 5.76; study 2: −5.17 to 4.19).

The differences in RR measurements are presented in Table 4. Approximately 90% of the measurement differences were within 5 measurements in studies 1 and 2.

## Discussion

This study evaluated the feasibility of monitoring HR and RR continuously using an NWD placed under the participant’s mattress and measured the consistency of the rates. The consistency of the HR and RR measured was proved by the finding that the mean differences in HR and RR calculated using the NWD in healthy participants and older adults who required nursing care were within the predetermined and accepted cutoffs. Moreover, none of the participants dropped out of the study or complained of physical abnormalities. Therefore, continuous monitoring of vital signs using NWDs is feasible at residences and in nursing homes.

First, no participants dropped out or complained in this study. Although a WD can provide accurate measurements, skin redness and itching have been reported due to skin contact with the device [9]; in contrast, our study participants did not report such skin-related issues. Moreover, if participants are aware that they are being monitored, their RR may tend to be lower than normal [35]. NWDs provide a nonintrusive alternative that allows for continuous monitoring in bed without skin contact or irritation [36]. In this study, an NWD yielded HR and RR values that were close to normal values because the patients were not uncomfortable and were less likely to notice that they were being monitored.

Second, we showed the correlation of HR measured using the NWD. The mean difference in HR measured using the NWD was within the predetermined acceptable range (study 1: −0.32 and 0.33 [nurse and WD, respectively] and study 2: 0.04 and 1.03 [nurse and WD, respectively]). In addition, the HRs measured with the NWD and WD were similar in this study. The mean differences in HR measured using other WDs are reportedly −0.20 (SD 5.54) [9], −1.1 (SD 3.8) [31], and 1.8 (SD 1.8) [37], close to those observed in this study. The device used in these previous studies was a wireless WD with electrodes attached to the anterior chest [9,31]. Although there are differences in measurement methods (ie, direct vs indirect contact with the skin), measurement positions (anterior chest vs posterior back), and device systems (electrical signals of the heart vs waves from body vibrations), the consistency in HR measurements suggests that both NWDs and WDs achieve similar accuracy. WDs can monitor an older adult’s activity continuously without much discomfort [36]. Both NWDs and WDs are noninvasive and can be easily integrated into the daily lives of older adults [38]. Therefore, it is important to select the appropriate device based on the specific needs and condition of the patient.

Third, we showed the correlation of RR measured by the NWD. The mean difference in the RR measured using the NWD was within the predetermined acceptable range (study 1: −0.98; study 2: −0.49). The mean differences in RR measured using other WDs are −2.3 (SD 6.8) [23] and 1.19 (SD 3.43) [9], similar to that in this study. We observed that the NWD could measure RR almost as well as the nurses, with a low measurement error (<5 breaths/min). However, a large difference of 17 breaths/min was seen in the measurement error (5 and 22 breaths/min for the nurse and NWD, respectively), consistent with a previous study by Weenk et al [9], who reported a difference of 26 breaths/min. In the present and previous studies, nurses assessed RR by visually observing the chest [9]. The reproducibility of the method is limited by high interobserver variability [39], and a large difference was expected in this study. In addition, the number of events related to the reliability of bradycardic and tachycardic respiration was low in this study. Therefore, their reliability could not be evaluated. The reasons for this include the short measurement time per participant, exclusion of participants with cardiac or respiratory disease, and starting the measurement after the participants had rested for 5 minutes. In the future, it is necessary to conduct long-term measurements, such as overnight measurements, to evaluate events related to the reliability of bradycardic and tachycardic respiration in older adults.

The strength of our study is that we evaluated the consistency of HR and RR measured using an NWD that has already been introduced in many facilities in Japan, and suggested the possibility of improving the nursing home environment. We believe that monitoring the HR and RR of older adults safely and unobtrusively when they are in bed may help in detecting sudden changes and providing suitable care without frequent visits. NWDs reduce the number of vital sign measurements and rounds and the burden on care providers, nurses, and care workers, allowing them to concentrate on care [40]. Given the declining population and limited number of health care workers, devices with consistent HR and RR will help optimize the patient’s environment. Therefore, we believe that NWDs can be applied in various facilities.

This study had some limitations. First, the participants were healthy or older adults without cardiac or respiratory disease. Therefore, the consistency in participants with cardiac and respiratory diseases should be examined further. Second, the measurement times for HR and RR in this study were short (5 minutes). When the NWD was installed at the residences and nursing homes, participants were continuously monitored while they were in bed. The disadvantages of continuous measurements, such as those taken overnight, were not considered. Third, the criterion measurement is lacking. In this study, nurse-based measurement was selected as the criterion for HR and RR as described in many studies [9,18,26]. Contact devices, such as electrocardiography, have also been used [8,9]. However, they are known to cause skin issues, such as peeling of the stratum corneum and red spots [41,42]. In this study, older adults (whose skin is typically more fragile) were recruited [13]. Therefore, we decided to use nurse-based measurement to prevent any discomfort and adverse events among the study participants. Ideally, HR and RR should have been measured using both methods. Despite these limitations, the consistency of HR and RR measured using the NWD suggests that this method may be useful for monitoring vital signs at residences and in nursing homes.

This study evaluated the feasibility and consistency of measuring HR and RR using an NWD placed under the mattress. The mean differences in HR and RR measured by the NWD were both within the predefined accepted discrepancies. No physical abnormalities were noted during the measurements. Therefore, we suggest using these devices at residences and in nursing homes. Safe monitoring of vital signs using NWDs is expected in the future for the early detection of abnormal conditions without inconveniencing older adults, care workers, and nurses. We believe that NWDs promote medical digital transformation, which will enable care providers to observe conditions accurately and provide appropriate care through the data obtained from such devices.

## Abbreviations

HR: heart rate
LoA: limit of agreement
NWD: nonwearable device
RR: respiratory rate
WD: wearable device

## References

[1] Borský P, Holmannová D, Fiala Z, Borská L, Hruška L, Kučera O (2022). Physiology of ageing. Cas Lek Cesk.

[2] Flint B, Tadi P (2023). Physiology, Aging.

[3] Celler BG, Sparks RS (2015). Home telemonitoring of vital signs--technical challenges and future directions. IEEE J Biomed Health Inform.

[4] Ashcraft AS, Owen DC (2014). From nursing home to acute care: signs, symptoms, and strategies used to prevent transfer. Geriatr Nurs (Minneap).

[5] Harris DA, Archbald-Pannone L, Kaur J (2021). Rapid telehealth-centered response to COVID-19 outbreaks in postacute and long-term care facilities. Telemed J E Health.

[6] Watkins T, Whisman L, Booker P (2016). Nursing assessment of continuous vital sign surveillance to improve patient safety on the medical/surgical unit. J Clin Nurs.

[7] Hollander JE, Carr BG (2020). Virtually perfect? Telemedicine for COVID-19. N Engl J Med.

[8] Kroll RR, Boyd JG, Maslove DM (2016). Accuracy of a wrist-worn wearable device for monitoring heart rates in hospital inpatients: a prospective observational study. J Med Internet Res.

[9] Weenk M, van Goor H, Frietman B (2017). Continuous monitoring of vital signs using wearable devices on the general ward: pilot study. JMIR Mhealth Uhealth.

[10] Boatin AA, Wylie BJ, Goldfarb I (2016). Wireless vital sign monitoring in pregnant women: a functionality and acceptability study. Telemed J E Health.

[11] Wang S, Ni X, Li L (2020). Noninvasive monitoring of vital signs based on highly sensitive fiber optic mattress. IEEE Sensors J.

[12] Wang Z, Yang Z, Dong T (2017). A review of wearable technologies for elderly care that can accurately track indoor position, recognize physical activities and monitor vital signs in real time. Sensors.

[13] Russell-Goldman E, Murphy GF (2020). The pathobiology of skin aging: new insights into an old dilemma. Am J Pathol.

[14] Ullal A, Su BY, Enayati M (2021). Non-invasive monitoring of vital signs for older adults using recliner chairs. Health Technol.

[15] de Tommasi F, Presti DL, Carassiti M, Schena E, Massaroni C Smart mattress based on fiber bragg grating sensors for respiratory monitoring: a feasibility test.

[16] Prata D, Carvalho A, Costa FM, Marques C, Leitao C (2021). Unobtrusive monitoring of the respiratory rate in an office desk chair with FBG sensors.

[17] Rohan R, Venkadeshwaran K, Ranjan P (2024). Recent advancements of fiber bragg grating sensors in biomedical application: a review. J Opt.

[18] (2015). World population ageing. United Nations.

[19] (2021). Dai 8 ki kaigo hoken zigyou keikaku ni motoduku kaigosyokuinn no hituyousuu nituite. Ministry of Health, Labour and Welfare.

[20] (2021). Kaigo robot no kaihatu fukyu no 488 sokushin. Ministry of Health, Labour and Welfare.

[21] (2015). Kaigo robot to donyu sien 491 tokubetu zigyou (Heisei 27 nendo hosei yosan). Ministry of Health, Labour and Welfare.

[22] (2020). Kaigo robbot no kaihatsu 494 hukyuu no sokushin. Ministry of Health, Labour and Welfare.

[23] Morley JE, Caplan G, Cesari M (2014). International survey of nursing home research priorities. J Am Med Dir Assoc.

[24] Sim I (2019). Mobile devices and health. N Engl J Med.

[25] A sensor to protect your loved ones. Safety Sheep Sensor.

[26] Steinhubl SR, Feye D, Levine AC, Conkright C, Wegerich SW, Conkright G (2016). Validation of a portable, deployable system for continuous vital sign monitoring using a multiparametric wearable sensor and personalised analytics in an Ebola treatment centre. BMJ Glob Health.

[27] Patel V, Orchanian-Cheff A, Wu R (2021). Evaluating the validity and utility of wearable technology for continuously monitoring patients in a hospital setting: systematic review. JMIR Mhealth Uhealth.

[28] Sun G, Matsui T, Watai Y (2018). Vital-SCOPE: design and evaluation of a smart vital sign monitor for simultaneous measurement of pulse rate, respiratory rate, and body temperature for patient monitoring. J Sens.

[29] Takahashi S, Yonekura Y, Takanashi N, Tanno K (2022). Risk factors of long-term care insurance certification in Japan: a scoping review. Int J Environ Res Public Health.

[30] Ikegami N (2019). Financing long-term care: lessons from Japan. Int J Health Policy Manag.

[31] Breteler Mm, Huizinga E, van Loon K (2018). Reliability of wireless monitoring using a wearable patch sensor in high-risk surgical patients at a step-down unit in the Netherlands: a clinical validation study. BMJ Open.

[32] Bland JM, Altman DG (1986). Statistical methods for assessing agreement between two methods of clinical measurement. Lancet.

[33] Bland JM, Altman DG (2003). Applying the right statistics: analyses of measurement studies. Ultrasound Obstet Gyne.

[34] Gerke O (2020). Reporting standards for a Bland-Altman agreement analysis: a review of methodological reviews. Diagnostics (Basel).

[35] Hill A, Kelly E, Horswill MS, Watson MO (2018). The effects of awareness and count duration on adult respiratory rate measurements: an experimental study. J Clin Nurs.

[36] Arandia N, Garate JI, Mabe J (2023). Monitoring of vital signs in the home environment: a review of current technologies and solutions.

[37] Hahnen C, Freeman CG, Haldar N (2020). Accuracy of vital signs measurements by a smartwatch and a portable health device: validation study. JMIR Mhealth Uhealth.

[38] Olmedo-Aguirre JO, Reyes-Campos J, Alor-Hernández G, Machorro-Cano I, Rodríguez-Mazahua L, Sánchez-Cervantes JL (2022). Remote healthcare for elderly people using wearables: a review. Biosensors (Basel).

[39] Edmonds ZV, Mower WR, Lovato LM, Lomeli R (2002). The reliability of vital sign measurements. Ann Emerg Med.

[40] Fulmer T, Reuben DB, Auerbach J, Fick DM, Galambos C, Johnson KS (2021). Actualizing better health and health care for older adults. Health Aff (Millwood).

[41] Feig DS, Donovan LE, Corcoy R (2017). Continuous glucose monitoring in pregnant women with type 1 diabetes (CONCEPTT): a multicentre international randomised controlled trial. Lancet.

[42] Ling Y, An T, Yap LW, Zhu B, Gong S, Cheng W (2020). Disruptive, soft, wearable sensors. Adv Mater.

